# The role of the microbiome in uterine cancer: insights into tumorigenesis, therapeutic implications, and clinical prospects

**DOI:** 10.1186/s43046-026-00372-9

**Published:** 2026-06-12

**Authors:** Yating Zhang, Xiaochuan Yu, Li Shi, Chen Qi, Huali Wang

**Affiliations:** 1 Dalian Medical University, Dalian, China; 2 Dalian Women and Children’s Medical Center (Group), Dalian, China

**Keywords:** Malignant tumors of the uterine cavity, Tumor microenvironment, Immune regulation, Early diagnosis, Prognosis assessment

## Abstract

Malignant tumors of the uterine cavity are a leading cause of cancer-related mortality in women worldwide. In recent years, the tumor microenvironment, as a novel biological concept, has garnered increasing attention. The microenvironment plays a crucial role in the initiation, progression, and response to treatment of tumors. Although research on the microenvironment of malignant uterine tumors is in its early stages, preliminary findings suggest that alterations in the microbial communities of cervical and endometrial cancers are closely associated with tumorigenesis, immune evasion, metastasis, and treatment resistance. Notably, specific microbial community changes have potential diagnostic and prognostic implications, offering novel insights into the management of these cancers.

This review aims to summarize and analyze the current state of research on the microenvironment of malignant uterine tumors, particularly its role in cervical and endometrial cancers. We first overview the epidemiological landscape and microenvironmental features of these tumors, emphasizing how microbial communities influence tumor initiation and progression. This influence occurs through immune modulation, metabolic changes, and alterations within the tumor microenvironment. Second, the potential role of the microenvironment in tumor therapy is explored, particularly its application prospects in chemotherapy, radiotherapy, and immunotherapy. In addition, the application potential of microbial communities in early tumor diagnosis, efficacy monitoring, and prognosis assessment is discussed.

## Introduction

### Epidemiology of malignant tumors of the uterine cavity

Malignant tumors of the uterine cavity remain a significant cause of cancer-related mortality in women worldwide. According to the latest statistics from the World Health Organization (WHO), the global incidence of cervical cancer in 2022 was approximately 660,000 cases, with an incidence rate of 14.12 per 100,000, making it the fourth most common cancer among women globally. Closely trailing cervical cancer is endometrial cancer, which ranked as the sixth most common cancer in women globally, with approximately 420,000 new cases diagnosed and an incidence rate of 8.4 cases per 100,000 women [1]. Zooming in on China, the statistics paint a concerning picture as well. According to the National Cancer Center of China’s 2024 report, cervical cancer accounted for approximately 150,700 new cases in 2022, with an incidence rate of 13.83 per 100,000 women. Meanwhile, the number of new cases of uterine corpus cancer, commonly referred to as endometrial cancer, stood at around 77,700, marking an incidence rate of 6.84 per 100,000 women. These figures highlight not only the high prevalence of these cancers in China but also a disturbing trend: the incidence of both cervical and endometrial cancers has been steadily increasing year on year, and they are affecting younger women [2]. Uterine sarcoma is a group of malignant tumors originating from the uterine smooth muscle, endometrial stroma, and/or connective tissues, accounting for 3%-7% of malignant tumors of the uterine cavity. In China, the incidence rate is even lower, standing at approximately 2 to 3 cases per 100,000 people [2]. It exhibits highly aggressive biological behavior, including early metastasis, high recurrence rates, and low 5-year survival rates. Choriocarcinoma is a highly malignant trophoblastic tumor characterized by early hematogenous metastasis, often secondary to pregnancy-related events. The incidence of choriocarcinoma is low. In China, it occurs in approximately 1 in 30,000 pregnancies [2].

### Emerging concepts in the tumor microenvironment

The concept of the tumor microenvironment (TME) has gained increasing attention in recent years as a key regulator of tumor initiation and progression. The TME refers to the composition and dynamics of microbial communities within the tumor and its microenvironment, including bacteria, viruses, fungi, and other microorganisms. Research indicates that the biological characteristics of tumors are no longer solely viewed as a result of host cell mutations but are closely associated with changes in the microbial communities within the TME [3]. The TME influences tumor initiation, immune evasion, metabolic reprogramming, and therapeutic responses through various mechanisms [4]. This concept has sparked extensive discussion on how microbial communities affect tumor biology, particularly in cancer immune evasion, drug resistance mechanisms, and cancer progression, where the role of microorganisms is becoming increasingly apparent.

The development of cervical cancer is closely linked to human papillomavirus (HPV) infection. Researchers have observed notable differences in the microbial population structure between cervical cancer patients and healthy individuals. These microbial variations may play a pivotal role in the persistence of HPV infections, ultimately influencing the onset and progression of cervical cancer [5]. Intriguingly, similar patterns emerge in endometrial cancer, where intrauterine microecological characteristics are associated with tumor behavior, including stage, metastasis, and recurrence. This suggests that the microbiota plays a significant role in the development and advancement of both cervical and endometrial cancers [6].

This review aims to delve into the potential role of microecology in uterine malignancies, particularly cervical and endometrial cancers, and to analyze its application prospects in tumor biology, treatment response, early diagnosis, and prognosis assessment. By synthesizing existing research findings, this article will explore the composition and changes of the microecology in uterine malignancies, and how the microecology promotes tumor occurrence and progression by affecting immune responses, metabolic pathways, and the remodeling of the tumor microenvironment, thereby revealing the potential of the microecology in tumor treatment, especially in immunotherapy, chemotherapy, and targeted therapy. Furthermore, the article scrutinizes the promising clinical applications of microbial communities as early diagnostic biomarkers and as tools for assessing tumor prognosis. As we peer into the future, this exploration anticipates the emerging trends and the intricate challenges that lie ahead in the domain of microecological research, painting a comprehensive picture of the intricate interplay between microecology and uterine malignancies.

## Normal state of the uterine cavity microbiota

### Composition of the uterine cavity microbiota

The composition of the uterine cavity microbiota in healthy women of reproductive age exhibits variability and diversity. The uterine cavity microbiota may be regulated by hormone levels and vary with changes in the menstrual cycle. Sola-Leyva et al. [7] performed a transcriptomic analysis of the endometrium from 7 healthy women, identifying specific bacterial species with high abundance in the uterine cavity, including *Streptococcus pneumoniae*,* Pasteurella multocida*,* Klebsiella pneumoniae*, and *Clostridium botulinum*. They posited that the uterine cavity microbiota differs across various phases of the menstrual cycle, with more transcriptionally active microorganisms (25 species) in the mid-secretory endometrium compared to the proliferative phase samples (8 bacteria), exhibiting higher transcriptional activity. This discovery challenged the prevailing notion that *Lactobacillus* dominates the uterine cavity, as they found *Lactobacillus* to be less prevalent at the transcriptionally active level in healthy young women [8, 9]. Pelzer et al. [10] proposed that *Prevotella spp*. is a marker of the proliferative phase, while *Sneathia spp*. is indicative of the secretory phase. Another study added depth to these findings by showing *Lactobacillus*’s prevalence varying throughout the menstrual cycle, being less abundant during menstruation, gradually increasing in the proliferative phase, and peaking during the secretory phase [11]. During the proliferative phase, species such as *Propionibacterium*,* Pseudomonas*, and *Sphingomonas* were found to be more abundant, further diversifying the uterine microbiota’s landscape [12]. However, Moreno et al. found no differences between bacterial communities in endometrial samples collected 2 and 7 days after the luteinizing hormone (LH) peak, suggesting that the endometrial microbiome does not appear to be influenced by hormonal changes [9]. Gressel GM et al. studied the uterine endometrial microbiota of postmenopausal women undergoing hysterectomy using 16 S rRNA sequencing and found that *Flavobacterium* was the primary genus in the uterine microbiota of postmenopausal women [13].

Wang et al. investigated the endometrial microbiome in 145 women, revealing a reduction in the diversity of the uterine cavity microbiome with advancing age, accompanied by an increase in the proportion of *Firmicutes* and a decrease in *Proteobacteria* [14]. Furthermore, the uterine cavity microbial ecology exhibited lower inter-individual variability in women aged 20 years and younger, whereas patients aged 41–60 years displayed the greatest inter-individual differences, suggesting an accumulation of disparities in the uterine cavity microenvironment with age.

### Physiological functions of the uterine cavity microenvironment

#### Anti-infective capacity

Uterine cavity lactobacilli are typically inversely correlated with pathogenic bacteria and positively correlated with commensal bacteria [15]. *Lactobacilli* produce metabolic products such as lactic acid, which creates an acidic environment that prevents pathogens like *Gardnerella vaginalis*,* Escherichia coli*, and *Cutibacterium acnes* from causing infections. *Bifidobacteria* possess the ability to synthesize organic acids and bacteriocin compounds; specifically, bifidocin produced by *Bifidobacteria* can inhibit the activity of pathogens such as *Listeria*,* Enterococcus*,* Pediococcus*,* Staphylococcus*,* Clostridium*,* Leuconostoc*,* and Bacillus strains* [16]. The extracellular polysaccharides produced by Lactobacilli act as microbe-associated molecular patterns, which interact with immune cells to stimulate the release of important immune-regulating factors like interleukin-12 (IL-12), tumor necrosis factor (TNF), interferon (IFN), transforming growth factor (TGF), and chemokines. This immune response helps to protect the uterine cavity against infection by foreign pathogens [17].

#### Regulation of uterine immune response

*Lactobacilli* represent the most prominent commensal bacteria within the reproductive tract of healthy women of reproductive age. *Lactobacillus crispatus* produces β-carboline compounds, which exhibit anti-inflammatory activity. These compounds reduce the production of pro-inflammatory cytokines such as TNF-α, IL-1β, and IL-6 by inhibiting the NF-κB signaling pathway, or by suppressing IFN signaling and significantly inhibiting the activation of type I IFN receptor (IFNAR) in monocytes, thereby suppressing B cell activity and exerting anti-inflammatory effects [18].*Lactobacillus crispatus* expresses an S-layer protein (SLP) that forms a 2D crystalline array on its cell surface. This array functions as a shield, protecting the bacteria from triggering TLR2-dependent pro-inflammatory pathways by shielding its own TLR2 ligands. Furthermore, this SLP selectively interacts with the anti-inflammatory receptor DC-SIGN, further enhancing its anti-inflammatory capabilities [19]. Lactic acid produced by lactobacilli can induce the secretion of the anti-inflammatory cytokine IL-10, reduce the production of the pro-inflammatory cytokine IL-12 in DCs, and reduce the cytotoxicity of NK cells [20]. In the context of immunomodulatory interactions, the genera *Lactobacillus* and *Sphingomonas* are positively correlated with DCs, NK cells, iTregs, and B cells, contributing to the clearance of pathogenic microorganisms [21]. *Bifidobacteria* alleviate inflammation by regulating the inflammatory signaling pathways JAK/STAT and NF-κB, and reducing the pro-inflammatory cytokines IL-6 and IL-1β [22].

#### Maintenance of pregnancy

*Lactobacilli* promote the proliferation of endometrial epithelial cells by modulating the phosphorylation of ERK1/2 proteins in the MAPK signaling pathway [23], which contributes to the repair and regeneration of the endometrium, thereby helping to improve the success rate of pregnancy. Moreno et al. found that polysaccharide A (PSA) expressed by *Bacteroides* in germ-free mice colonized with *Bacteroides fragilis* strain NCTC9343 increases the CD4 + population and activates signal transduction linked to TLR2, leading to Th1 cell differentiation and the establishment of an appropriate Th1/Th2 balance, which plays a role in endometrial receptivity [24], and is conducive to placental implantation. The composition of the endometrial microbiota can predict pregnancy outcomes. A high abundance of *Lactobacillus* in the endometrium is conducive to better reproductive outcomes, whereas a depletion of Lactobacillus leads to an increase in pathogenic microorganisms such as *Atopobium*,* Bifidobacterium*,* Chryseobacterium*,* Gardnerella*,* Haemophilus*,* Klebsiella*,* Neisseria*,* Staphylococcus*, and *Streptococcus.* These pathogenic microorganisms will stimulate the innate immune response of endometrial cells, produce inflammatory factors, and cause chronic endometritis, leading to adverse reproductive outcomes of clinical abortion or no pregnancy [15].

## Microbiome research in uterine malignancies

Imbalance of the uterine cavity microbiota is often manifested as a decrease in the number of *lactobacilli* or overgrowth of pathogenic microorganisms. Studies have shown that the decrease in *Lactobacillus* levels is closely related to the occurrence of various uterine malignancies, suggesting that the imbalance of the micro-ecology may be a potential promoting factor for tumor development. Dysbiosis of the uterine microbiota may result in a decrease in beneficial lactobacilli or an overgrowth of harmful pathogens. Studies have shown that reduced levels of lactobacilli are associated with the development of uterine cancers. This implies that an unhealthy microbial ecology might potentially promote tumor growth. Therefore, maintaining a healthy balance of bacteria in the uterus could be essential for preventing the development of malignancies.

### Endometrial Cancer (EC)

Research employing 16 S rDNA, 16 S rRNA, and metagenomic sequencing has revealed a significant enrichment of specific bacterial phyla within the endometrium of endometrial cancer patients. These include *Firmicutes*,* Proteobacteria*,* Tenericutes*,* Actinobacteria*,* and Bacteroidetes;* with notable enrichment at the genus level observed for *Porphyromonas*,* Prevotella*,* Helicobacter*,* and Peptoniphilus* [25–27]. Walther et al. [25] analyzed endometrial samples from EC patients and those with benign uterine conditions using 16 S rDNA V3-V5 region sequencing. Their findings indicated that *Staphylococcus*,* Blautia*, and *Paraprevotella* predominated in the benign cohort, whereas *Bacteroides* and *Faecalibacterium* were enriched in the EC cohort. Furthermore, *Atopobium vaginae* and *Porphyromonas sp.* were associated with the presence of EC under conditions of vaginal pH > 4.5. Peigen Chen et al. [26] utilized metagenomic sequencing and identified the top five enriched phyla in the endometrium of EC patients as *Firmicutes*,* Proteobacteria*,* Tenericutes*,* Actinobacteria*, and *Bacteroidetes*. Meanwhile, a study by Semertzidou et al. [27] focused on characterizing the microbial composition in the vagina, cervix, and endometrium of endometrial cancer patients using 16 S rRNA gene sequencing. They observed a high degree of microbial diversity in the vagina, cervix, and endometrium of EC patients, with a marked reduction in the abundance of *Lactobacillus.* Enrichment was noted for *Anaerococcus*,* Porphyromonas*,* Prevotella*,* Fusobacterium*,* Bacteroides*, and *Peptostreptococcus.* Subsequently, Semertzidou et al. used 3D benign and endometrial cancer organoids to assess the impact of microbial products. They found that microbial products from Lactobacillus crispatus exhibited resistance to endometrial cell proliferation, with a lesser impact on cytokine/chemokine profiles. Wang et al. [28] reported increased abundance of *Prevotella*,* Atopobium*,* Anaerococcus*,* Dialister*,* Porphyromonas*, and *Peptostreptococcus* in the endometrium of EC patients.

### Cervical Cancer (CC)

Recent research has shed light on the correlation between certain bacteria and the presence of cervical dysplasia or cancer in women. In a unique study, *Sneathia* and *Fusobacterium spp*. were found exclusively in the cervix of women with these conditions, while absent in those without tumors. *Sneathia spp.* are characteristic of the cervical microbiota in patients with HPV-positive squamous intraepithelial lesions, whereas *Fusobacterium spp.* are indicative of cervical cancer patients [29]. Zhou et al. [30], through retrospective and prospective cohort analyses, identified Escherichia coli as the predominant pathogen isolated from cervical secretions of CC patients, followed by *Klebsiella pneumoniae*,* Proteobacteria*, and *Enterococcus faecalis.*

## Mechanisms of the tumor microenvironment in uterine malignancies

### Microbe-driven immune responses

Microbe-mediated inflammatory responses: Chronic microbial infections or dysbiosis can trigger chronic inflammatory responses. Chronic inflammation is considered a significant risk factor for tumorigenesis, potentially promoting tumor development by altering the cell cycle, inducing gene mutations, or through immune evasion mechanisms. Lu et al. [31] observed an increase in *Micrococcus* in the uterine cavity microbiota of endometrial cancer (EC) patients, with the relative abundance of *Micrococcus* positively correlated with IL-6 and IL-17 mRNA levels. L-6 is known to play a role in promoting the growth of EC cells by activating the ERK-NF-κB signaling pathway, creating a feedback loop that stimulates tumor growth [32]. Similarly, IL-17 activates different signaling pathways in EC cells, leading to the production of pro-inflammatory cytokines, chemokines, antimicrobial peptides, and matrix metalloproteinases. This alteration of the endometrial microenvironment can facilitate the growth of tumor cells [33].Furthermore, the presence of *Micrococcus* bacteria can increase the signaling of the DUOX2 oxidase in tumor cells. DUOX2 expression has been linked to increased tumor growth potential and poor prognosis in cancer patients [34]. Zhang et al. [35] found that extracellular superoxide produced by *Enterococcus faecalis* increased the expression of TNFα, IL1B, and IL6 inflammatory cytokines in EC cells, while downregulating the expression of endometrial receptivity-related gene markers such as integrin αV, integrin β3, LIF, and HB-EGF. This dual effect—inducing inflammation while reducing endometrial receptivity—creates an environment that is hostile to normal endometrial function and permissive to EC development. Additionally, it promotes the apoptosis of endometrial epithelial cells by decreasing the levels of anti-apoptotic proteins Bcl-2 and MCL-1. Lipopolysaccharides (LPS), produced by bacteria such as *Atopobium* and *Porphyromonas* in the uterine cavity of EC patients, interact with macrophages via Toll-like receptor 4 (TLR4). This interaction induces macrophage M1 polarization and the production of pro-inflammatory cytokines TNF-α, IL-1α, IL-1β, IL-6, and IL-12 [27, 36, 37]. These cytokines amplify the inflammatory response, further exacerbating the tumor-permissive environment. *Prevotella* is another microorganism that plays a significant role in EC by augmenting IL-17 secretion. It promotes Th17 cell differentiation, leading to increased IL-17 levels [38]. Overexpression of IL-1α and IL-1β in various tumors, including EC, leads to elevated expression of interleukin-1 receptor-associated kinase 1 (IRAK1). This IRAK1 overexpression activates cell cycle-related factors such as CDK1, Cdc452, and cell division pathway-related factors Cdc7 and MCM222, thereby promoting abnormal cell proliferation and EC tumorigenesis [39]. TNFα induces increased expression of CCL8 and CXCL2 in endometrial cancer. CCL8 upregulates the sialic acid-binding receptor SIGLEC1 on tumor-associated macrophages (TAMs). Co-expression of SIGLEC1 and CCL8 serves as a prognostic marker for poor survival, while CXCL2 induces chemoresistance [40]. IL-17 may enhance endometrial cancer cell proliferation and migration by upregulating the estrogen receptor α (ERα)/ERβ ratio in these cells [41]. The enrichment of *Fusobacterium* in the uterine cavity is associated with the infiltration of regulatory T cells (Tregs) [27, 42]. Tregs suppress anti-tumor immune responses by secreting inhibitory cytokines TGF-β and IL-10, thereby promoting immune evasion of the tumor [43]. The metabolism of the endometrial microbiome also contributes to EC development. Han et al. [44] discovered a notable correlation between *Penicillium* and EC, indicating that *Penicillium* may contribute to the reactivation of estrogen through the secretion of β-glucuronidase, thereby influencing circulating estrogen levels. Estrogen promotes EC cell proliferation and migration through GPER-mediated MEK/ERK/MAPK and PI3K/AKT signaling pathways, further contributing to EC progression. Bai et al. [45] discovered that *Porphyromonas* can induce the expression of inflammatory genes IL-1β, CXCL8, and JUN in cervical epithelial cells. IL-1β significantly increases the proliferation and tumorigenic potential of cervical epithelial cells, thereby promoting the development of cervical cancer.

### Interactions between microbes and tumor genomics

Dalmasso G et al. [46] posited a link between *Escherichia coli* and cancer progression, wherein *E. coli*, under miRNA control, disrupts protein SUMOylation by downregulating SENP1, thereby driving cellular senescence. Senescent cells, in turn, stimulate tumor growth by producing growth factors such as HGF, FGF, and GM-CSF. *Firmicutes*, through the production of butyrate, induce histone acetylation, leading to chromatin relaxation and promoting the transcriptional activation of the *p21* gene. The *p21* protein prevents cell cycle progression from the G1 phase to the S phase, causing cell cycle arrest in the G1 phase and promoting cancer cell apoptosis, thus exerting an anti-cancer effect [47]. HPV has been demonstrated to be closely associated with the development of cervical cancer. HPV interferes with the host cell genome through its oncogenic proteins, E6 and E7, which promote abnormal cell proliferation and carcinogenesis. The E6 oncoprotein primarily exhibits tumor effects by promoting the ubiquitin-dependent proteasomal degradation of p53 in HPV-infected cells, thereby evading cell death [48]. Furthermore, E6 also targets the degradation of other apoptotic signaling cascade molecules, such as Bak, Fas-associated death domain (FADD), and Bax, via the ubiquitin-proteasome pathway, which inhibits apoptosis and contributes to tumor development [49]. The pRB protein is known to halt the cell cycle by inhibiting E2F factor-induced unlimited cell division and cancer progression. E7, by degrading the pRB protein, causes cell cycle dysregulation, thereby leading to cancer progression [50].

## Microecology and therapeutic response in malignant tumors of the uterine cavity

### Synergistic effects of microecology and immunotherapy

*Lactobacilli* are the most representative commensal bacteria in the uterine cavity of healthy women of childbearing age. They can induce the secretion of the anti-inflammatory cytokine IL-10, reduce the production of the pro-inflammatory cytokine IL-12 in dendritic cells, and reduce the cytotoxicity of NK cells [20], thereby reducing inflammation and tumor formation, becoming a new approach to tumor immunotherapy [51]. Jia et al. found that the abundance of *Lactobacillus johnsonii* in commensal *lactobacilli* was positively correlated with the responsiveness of immune checkpoint blockade (ICB) in human cancers. *L. johnsonii* cooperates with *Clostridium sporogenes* to produce indole-3-propionic acid (IPA). IPA regulates the stem cell program of CD8 T cells by increasing H3K27 acetylation in the Tcf7 super-enhancer region and promotes the production of progenitor-exhausted CD8 T cells (T+++pex) to improve the therapeutic effect of ICB. *L. johnsonii* and bacterial-derived IPA provide new adjuvant therapeutic approaches for the study of malignant tumors of the uterine cavity [52]. In recent years, immune checkpoint inhibitors (ICIs) have become an important therapeutic strategy for endometrial cancer. PD-1/PD-L1 inhibitors are a type of immune checkpoint inhibitor that activates the immune response of T cells against tumor cells by disrupting tumor immune escape mechanisms [53]. The microbiota plays a key role in regulating the efficacy of immune checkpoint inhibitor therapy. Sivan [54] et al. reported the association between the microbiota and the efficacy of ICIs, emphasizing that signals derived from the genus *Bifidobacterium* regulate the activation of dendritic cells under homeostasis, which in turn supports the improvement of tumor-specific CD8 + T cell effector function, not only promoting anti-tumor immunity but also promoting the efficacy of anti-PD-L1.

### Prospects for the application of microbiome modulation strategies

In cervical cancer, probiotics have demonstrated diverse anti-cancer effects, including the inhibition of cell viability, suppression of metastasis, reduction of proliferation, and induction of apoptosis. Wang et al. demonstrated that *Lactobacillus* supernatant could limit the proliferation of cervical cancer CaSki cells; after treatment with the supernatant of *Lactobacillus*, the number of cells in the S phase markedly rose, while the number of cells in the G2/M phase declined. Furthermore, Lactobacillus supernatant reduced the expression of cyclin A, CDK2, and E6/E7 HPV oncogenes, which are essential for malignant transformation [55]. In the treatment of cervical cancer, Negi et al. suggested that a pessary containing probiotic biomass and cisplatin might be a more effective method for treating cervical cancer [56]. These probiotics include commonly used strains such as *Lactobacillus rhamnosus GG (LGG)*,* Lactobacillus acidophilus*,* Bifidobacterium*, and *Saccharomyces boulardii*, which have positive effects such as scavenging free radicals and may also have the ability to enhance anti-tumor effects [57]. Some studies have found that the gut microbiota of patients with advanced rectal cancer is dominated by *Lactobacillus paracasei*,* Porphyromonas*, and *Lactobacillus fermentum.* These microorganisms drive the biosynthesis of GDP-D-rhamnose and butyrate, significantly affecting the response of patients with advanced rectal cancer to neoadjuvant chemoradiotherapy, providing valuable insights for improving the benign prognosis probability of neoadjuvant chemoradiotherapy [58, 59]. Although research on the intrauterine microenvironment in the context of malignant tumors is lacking, it presents an intriguing avenue for future investigation.

## The microbiome as a diagnostic and prognostic biomarker for malignant tumors of the uterine cavity

### Microbiome and early tumor detection

In recent years, the tumor-associated microbiome has been proposed as a potential early diagnostic biomarker. Specifically, in cervical cancer research, evidence has emerged linking specific microbial communities to tumor development. Wu et al. [60] analyzed the cervical microbiome of patients at different stages of cervical cancer using 16 S rDNA analysis. The results revealed that the relative abundance of *Lactobacillus* was lower, while the relative abundance of anaerobic bacteria was higher in more severe cervical lesions. They determined that certain genera serve as markers for cervical cancer, such as *Porphyromonas*,* Prevotella*, and *Campylobacter*. Additionally, the genus *Corynebacterium* was identified as a marker for high-grade squamous intraepithelial lesions. Liu et al. [6] found that *Actinobacteria* and *Bacteroidetes* were dominant in the uterine cavity in EC, suggesting that bacteria of the genus *Blautia* may serve as a uterine cavity microbial marker for EC patients. Kuźmycz et al. [61] identified *Peptoniphilus* as a potential uterine cavity microbial marker for EC. Walsh et al. [62] determined that the presence of *Porphyromonas somerae* is the most predictive uterine cavity microbial marker for EC, aiding in the detection of EC in high-risk, asymptomatic women.

### The microbiome and tumor prognosis

Zhou et al. [63] suggested that higher levels of Micrococcus in the tumor microenvironment are associated with a worse prognosis in patients with human papillomavirus-independent endometrial adenocarcinoma (HPVI ECA), whereas elevated levels of Streptococcus are linked to reduced recurrence-free survival in these patients. Liu et al. [6] found that a decline in the abundance of 20 microbial markers, mainly including the genera *Nitratireductor*, *Campylobacter*,* Limnohabitans*,* and Cyclobacterium*, is related to poorer outcomes in endometrial cancer (EC) patients. Conversely, an increase in the abundance of six microbial markers, predominantly *Elizabethkingia* and *Blautia* genera, was associated with unfavorable prognoses in EC patients. Recent investigations have revealed a significant correlation between the uterine microbiome and overall survival (OS) in EC patients, identifying nine microbial genera as independent factors influencing OS. Genera associated with favorable prognosis included *Zooshikella*,* Caldimonas*,* Candidatus Accumulibacter*,* Nonlabens*,* Roseiflexus*,* Streptosporangium*,* and Zavarzinella*, whereas *Derxia* and *Shigella* were linked to adverse outcomes [64].

## Conclusion

Research into the tumor microenvironment in endometrial malignancies offers novel perspectives, facilitating a deeper understanding of the mechanisms underlying tumor initiation, progression, and therapeutic response. The microenvironment plays a pivotal role not only in tumorigenesis but also significantly influences immune evasion, treatment resistance, and prognostic assessment. Through refined microenvironmental investigations, the development of novel early diagnostic biomarkers, therapeutic targets, and prognostic tools is anticipated, thereby advancing early detection and personalized treatment strategies for endometrial malignancies.

As technology continues to advance, especially with the use of high-throughput sequencing and artificial intelligence, the study of the microenvironment in cancer research is becoming increasingly important. Future endeavors, characterized by interdisciplinary collaboration, meticulous research, and the promotion of clinical translation, hold the potential for the microenvironment to emerge as a crucial tool in the diagnosis and treatment of endometrial malignancies, ultimately providing patients with more precise and individualized therapeutic approaches.

## Data Availability

No datasets were generated or analysed during the current study.

## Abbreviations

HPV: Human papillomavirus
TNF: Tumor necrosis factor
EC: Endometrial cancer
CC: Cervical cancer
